# Kidney biopsy diagnosis in childhood in the Norwegian Kidney Biopsy Registry and the long-term risk of kidney replacement therapy: a 25-year follow-up

**DOI:** 10.1007/s00467-022-05706-y

**Published:** 2022-08-22

**Authors:** Ann Christin Gjerstad, Rannveig Skrunes, Camilla Tøndel, Anders Åsberg, Sabine Leh, Claus Klingenberg, Henrik Døllner, Clara Hammarstrøm, Anna Kristina Bjerre

**Affiliations:** 1 Division of Pediatric and Adolescent Medicine, Oslo University Hospital, Oslo, Norway; 2 Department of Medicine, Haukeland University Hospital, Bergen, Norway; 3 Department of Clinical Medicine, University of Bergen, Bergen, Norway; 4 Department of Clinical Science, University of Bergen, Bergen, Norway; 5 Department of Pediatrics, Haukeland University Hospital, Bergen, Norway; 6 The Norwegian Renal Registry, Oslo University Hospital – Rikshospitalet, Oslo, Norway; 7 Department of Transplantation Medicine, Oslo University Hospital – Rikshospitalet, Oslo, Norway; 8 Department of Pharmacy, University of Oslo, Oslo, Norway; 9 Department of Pathology, Haukeland University Hospital, Bergen, Norway; 10 Department of Pediatrics and Adolescence Medicine, University Hospital of North Norway, Tromsø, Norway; 11 Paediatric Research Group, Faculty of Health Sciences, UiT-The Arctic University of Norway, Tromsø, Norway; 12 Department of Clinical and Molecular Medicine, Norwegian University of Science and Technology (NTNU), Trondheim, Norway; 13 Children’s Clinic, St. Olavs Hospital, Trondheim University Hospital, Trondheim, Norway; 14 Department of Pathology, Oslo University Hospital - Rikshospitalet, Oslo, Norway; 15 Institute of Clinical Medicine, University of Oslo, Oslo, Norway

**Keywords:** Kidney biopsy, Chronic kidney disease stage 5, Kidney replacement therapy, National registries, Children

## Abstract

**Background:**

There is scarce information on biopsy-verified kidney disease in childhood and its progression to chronic kidney disease stage 5 (CKD 5). This study aims to review biopsy findings in children, and to investigate risk of kidney replacement therapy (KRT).

**Methods:**

We conducted a retrospective long-term follow-up study of children included in the Norwegian Kidney Biopsy Registry (NKBR) and in the Norwegian Renal Registry (NRR) from 1988 to 2021.

**Results:**

In total, 575 children with a median (interquartile range, IQR) age of 10.7 (6.1 to 14.1) years were included, and median follow-up time (IQR) after kidney biopsy was 14.3 (range 8.9 to 21.6) years. The most common biopsy diagnoses were minimal change disease (MCD; *n* = 92), IgA vasculitis nephritis (IgAVN; *n* = 76), IgA nephropathy (*n* = 63), and focal and segmental glomerulosclerosis (FSGS; *n* = 47). In total, 118 (20.5%) of the biopsied children reached CKD 5, median (IQR) time to KRT 2.3 years (7 months to 8.4 years). Most frequently, nephronophthisis (NPHP; *n* = 16), FSGS (*n* = 30), IgA nephropathy (*n* = 9), and membranoproliferative glomerulonephritis (MPGN; *n* = 9) led to KRT.

**Conclusions:**

The risk of KRT after a kidney biopsy diagnosis is highly dependent on the diagnosis. None of the children with MCD commenced KRT, while 63.8% with FSGS and 100% with NPHP reached KRT. Combining data from kidney biopsy registries with registries on KRT allows for detailed information concerning the risk for later CKD 5 after biopsy-verified kidney disease in childhood.

**Graphical abstract:**

A higher resolution version of the Graphical abstract is available as Supplementary information

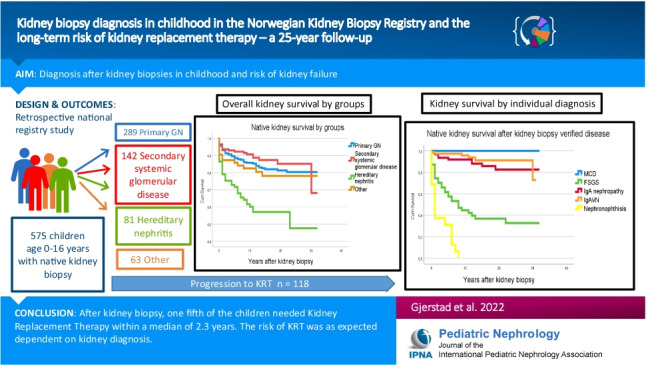

**Supplementary Information:**

The online version contains supplementary material available at 10.1007/s00467-022-05706-y.

## Introduction

Kidney biopsy is the gold standard for diagnosing kidney disease. Kidney biopsy is a safe procedure [1] that supports correct diagnosis and hence the need for prompt or preventive treatment. The core question in preventive, diagnostic, and therapeutic nephrology is how many individuals develop chronic kidney disease stage 5 (CKD 5) ultimately needing kidney replacement therapy (KRT).

Kidney biopsy registries capture most glomerulonephritis in childhood including those who proceed to KRT. In addition, a KRT registry will include congenital anomalies of the kidney and urinary tract (CAKUT) and different hereditary nephropathies including nephronophthisis (NPHP) that are responsible for 34–43% and 19–34% of pediatric KRT, respectively [2–5].

A more comprehensive overview of kidney disease outcome is achievable by linking biopsy data with data on progression to CKD 5.

The primary aim of the study was to present, based on data from a national kidney biopsy registry with the support from clinical findings and genetics, comprehensive data on biopsy-verified kidney disease in children aged 0–15 years. The secondary aim was to evaluate how many of these children, and which diagnoses, led to progressive kidney disease and need of KRT in the long term.

## Subjects and methods

### Registries

The Norwegian Kidney Biopsy Registry (NKBR) registers information on pathology data from native kidney biopsies and selected clinical data. Norwegian Renal Registry (NRR) is a national quality registry including patients in all age groups treated with KRT. Inclusion into the NKBR and NRR is based on informed, written consent by the patient and/or their designees. A few regional hospitals perform pediatric kidney biopsies and Oslo University Hospital performs all transplantations in Norway, which gives the registries nearly complete follow-up possibilities. NNR, but not NKBR, does not record refusal of inclusion.

All patients below 16 years of age registered in NKBR from March 1, 1988 to December 31, 2013 were included. We merged data from NKBR with information from the NRR to identify patients who received KRT until December 31, 2020. If the reports in NKBR were insufficient, we searched the journal for supplementary information regarding biopsy data and diagnosis. If the information still was lacking, the patients were excluded. All children with dialysis as the bridge to transplantation were included; the start of KRT was set at the time of starting dialysis or the day of transplantation. To assess for changes in the different diagnoses over time in NKBR, we compared an early period (1990–1994) with a late period (2009–2013).

### Classifications

We grouped the kidney diagnosis according to Hou et al., modified to include inherited tubulopathies in hereditary disease [6]. Hereditary kidney disease in a childhood dataset contains various syndromes and hereditary kidney diseases (Table 1). NPHP, polycystic kidney disease, and other ciliopathies are gathered under the term renal ciliopathies [7]. Focal segmental glomerulosclerosis (FSGS) in children frequently has a genetic background, but we chose to use the traditional classification considering FSGS as a primary glomerular disease. The term IgA vasculitis nephritis (IgAVN) was preferred to Henoch Schönlein Purpura nephritis [8]. We used the term mesangioproliferative glomerulonephritis (MesPGN) in mesangioproliferative glomerulonephritis without IgA deposits and no other manifestations consistent with another kidney disease or infection. Calcineurin inhibitor (CNI) toxicity as diagnosis only reflects children with another cause for use of calcineurin inhibitor rather than kidney disease. Biopsy-proven CNI toxicity connected to treatment for primary GN like MCD was thus classified as MCD and not CNI toxicity.

**Table 1:** Classification of kidney disease after clinic pathological origin, sex, time periods, and connection to KRT

	Biopsy					KRT					
	Boys	Girls	Median age, range	1990–1994	2009–2013	Boys	Girls	Median age, range	1990–1994	2009–2013	Total 1988–2020 (%)
Primary glomerular disease	163	126	10 (0.3–16)	41	62	28	23	14.6 (4.1–45)	7	9	51 (17.6)
Secondary systemic glomerular disease	70	72	11.3 (0.1–16)	16	48	9	10	14 (2.3–31.4)	5	1	19 (5.3)
Hereditary kidney disease	43	38	9 (0.1–15.8)	7	19	15	21	12.8 (0.2–38.6)	4	9	36 (44.4)
TIN	17	15	12.7 (0–15.9)	2	12	0	0		0	0	0 (0.0)
Unclassified GN^*^	6	3	10.4 (2.4–15.6)	3	0	2	1	14.8 (6.6–23.3)	0	0	3 (50.0)
Normal^*^	6	3	13.4 (0–15.8)	1	0	0	0		0	0	0 (50.0)
CAKUT^*^	4	2	5.3 (0.4–14.9)	1	2	1	2	19.3 (9.3–24.3)	0	1	3 (50.0)
CKD 5^*^	4	2	13.4 (12.8–15.1)	2	1	4	2	13.7 (12.8–15.1)	2	1	6 (100.0)

Primary glomerular diseases: post-infectious GN (PIGN), minimal change disease (MCD), membranous GN, mesangioproliferative GN (MesPGN), membranoproliferative GN (MPGN), focal and segmental glomerulosclerosis (FSGS), IgA nephropathy

Secondary systemic glomerular diseases: lupus nephritis, ANCA glomerulonephritis (ANCA GN), anti-glomerular basement membrane disease (anti-GBM), IgA vasculitis nephritis (IgAV nephritis), amyloidosis, nephrosclerosis

Hereditary renal diseases: Alport syndrome, thin basement membrane disease (TBMD)

Metabolic diseases: nephronopthisis (NPHP), other ciliopathies, congenital nephrotic syndrome (CNS), tubulopathies

Tubulointerstitial diseases: acute tubulointerstitial nephritis (acute TIN), chronic tubulointerstitial nephritis (chronic TIN), acute tubular necrosis (ATN), calcineurin inhibitor toxicity

^*^Unclassified GN, normal biopsy, CAKUT, and chronic kidney disease stage 5 (CKD 5) were added as diagnostic groups

## Statistics

We applied SPSS Statistics 26 for statistical analysis and used descriptive statistics to describe the different diagnoses and the respective long-term outcomes. The chi-square test was used for comparison between observed results and *p* < 0.05 was considered statistically significant. Interquartile range (IQR) defined the difference in spread between the 25th and 75th percentiles in age at biopsy and over all follow-up time. We used Kaplan–Meier plot for survival curves. The population below 16 years in Norway between 1990 to 1994 and 2009 to 2013 was found in Statistics Norway (07,459: Population, by year and contents. Statbank Norway (ssb.no).

## Ethical approval

The Regional Ethics Committee of Eastern Norway (2014/2319/REK) approved the study including approval to review medical files and if necessary, to clarify the diagnosis.

## Results

We identified 586 children with a kidney biopsy of which 575 children were included, 313 (54.4%) boys. Median (IQR) age at biopsy was 10.7 (6.1 to 14.1) years, and the median (IQR) follow-up time after kidney biopsy was 14.2 (8.9 to 21.6) years.

Primary glomerular disease was the predominant category in the kidney biopsies (*n* = 289, 50.3%), followed by secondary systemic glomerular disease (*n* = 142, 24.7%). See Table 1 for a complete overview of the distribution of the different groups.

**Fig. 1:**
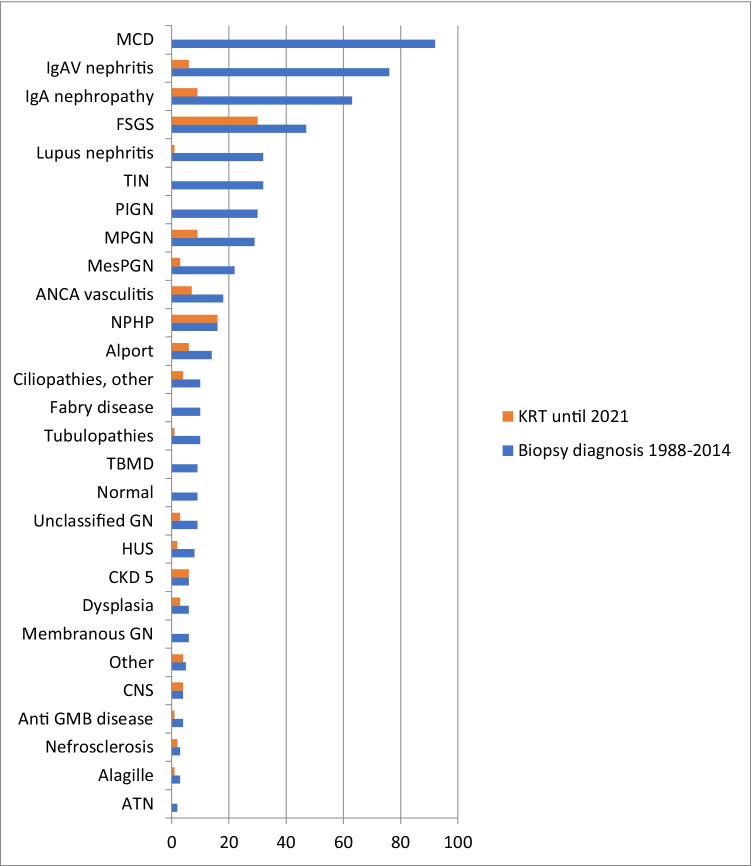
Number of kidney biopsy diagnoses in children in the time period 1988 to 2014 and number that reached KRT within 2021. *ATN* acute tubular necrosis, *CNS* congenital nephrotic syndrome, *CKD 5* chronic kidney disease stage 5, *FSGS* focal segmental glomerulosclerosis, *GN* glomerulonephritis, *HUS* hemolytic uremic syndrome, *MCD* minimal change disease, *MesPGN* mesangioproliferative glomerulonephritis, *MPGN* membranoproliferative glomerulonephritis, *NPHP* nephronophthisis, *PIGN* post-infectious glomerulonephritis, *TBMD* thin basement membrane disease, *TIN* tubulointerstitial nephritis

**Fig. 2:**
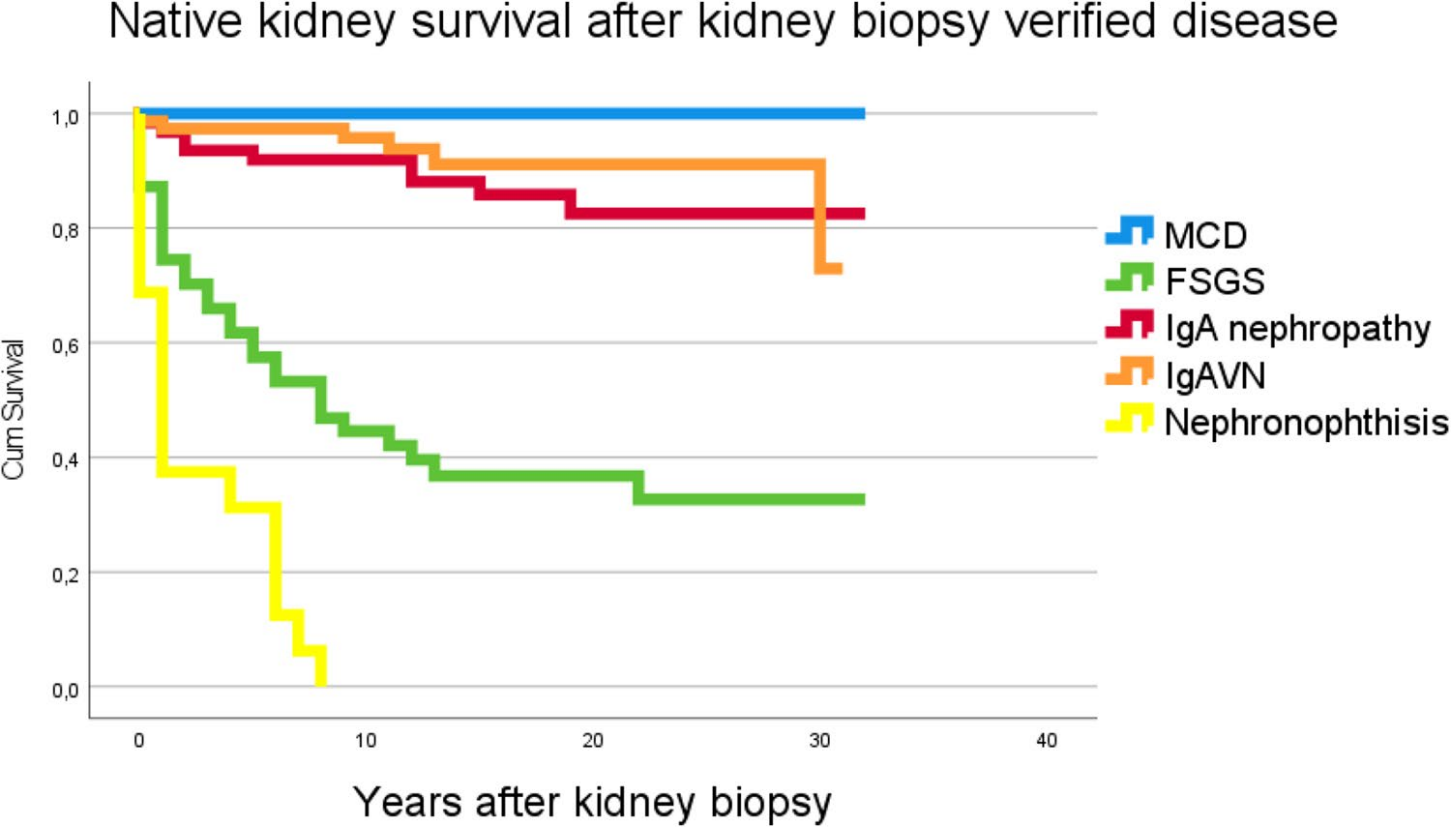
Kidney survival after kidney biopsy verified MCD, FSGS, IgA nephropathy, IgAVN, and nephronophthisis

Minimal change disease (MCD) was the most common histological diagnosis (*n* = 92, 16.0%), followed by IgAVN (*n* = 76, 13.2%), IgA nephropathy (*n* = 63, 11%), and FSGS (*n* = 47, 8.2%). See Fig. 1 for a complete report. Shown in Fig. 2 are survival curves for some of the kidney diseases.

In total, 118 children with a biopsy-confirmed diagnosis reached KRT (20.5%), 59 boys (50%), with a median (IQR) time to KRT of 2.3 (7 months to 8.4) years (Fig. 1). The risk for commencing KRT was greatest for hereditary kidney disease (44.4%) and primary glomerular disease (27.6%) (Table 1). Of the specific diagnoses, all children with NPHP reached KRT (100%) followed by FSGS (63.8%) (Table 2). Similar proportions of children with IgA nephropathy and IgAVN reached KRT: IgA nephropathy 14.5% KRT (median age 20.9 years, range 12.5–33.1 years) and IgAVN 7.9% KRT (median age at KRT 22.2 years, range 8.8–45.9 years), *p* = 0.21. Table 2 shows details for common biopsy diagnoses leading to KRT.

**Table 2:** Biopsy diagnosis in children in need of KRT

	Biopsy			KRT				
	*N*	Median age in years (range)	Male (%)	*N*	< 16 years	Median age in years at start KRT (range)	Male (%)	Total reaching KRT (%)
FSGS	47	8.3 (0.4–15.9)	51.1	30	21	13.9 (4.2–37.8)	56.7	63.8
NPHP	16	8.3 (2.7–15.8)	50.0	16	15	13.1 (2.8–16.2)	50.0	100.0
IgA nephropathy	63	14.2 (3.7–15.9)	63.5	9	3	20.9 (12.5–33.1)	77.8	14.5
MPGN	29	9.1 (2.3–15.9)	44.8	9	3	16.8 (4.3–23.7)	44.4	31.0
ANCA GN	18	12.9 (2.5–15.6)	38.9	7	5	13.5 (5.4–26.3)	42.9	38.9
IgAVN	76	9.4 (2.6–15.9)	59.2	6	2	22.2 (8.8–45.9)	50.0	7.9
Alport syndrome	13	10.4 (5.0–15.3)	69.2	6	0	22.7 (17.1–35.8)	50.0	46.2
MesPGN	22	11.6 (0.7–15.7)	31.8	2	2	13.5 (11.7–15.3)	0.0	13.6

*N* number of patients

There were only 8 patients that had not commenced kidney transplantation in NRR, of which 4 had died while on dialysis, with the remaining 4 still waiting for transplantation.

The number of kidney biopsies were 1.6/100,000 per year in children below 16 years from 1990 to 1994 and 2.9/100,000 per year between 2009 and 2013, but there were no significant differences in distribution across time (primary kidney disease *p* = 0.68, secondary systemic glomerular disease *p* = 0.08, and hereditary kidney disease *p* = 0.4). IgAVN was the only diagnosis that increased in frequency across time. For comparison of the four most common diagnosis between 1990 to 1994 and 2009 to 2013, see Table 3. There was no major increase in number of patients reaching KRT between 1990 to 1994 and 2009 to 2013, 18 and 21 patients, respectively. Due to small numbers, it was not possible to ascertain temporal changes for time to KRT in individual diagnoses.

**Table 3:** Comparison between the four most frequent biopsy diagnoses between 1990 to 1994 and 2009 to 2013

	1990–1994 children (%)	2009–2013 children (%)	*p*-Value
Total	73	144	
MCD	12 (16.4)	24 (16.7)	0.966
FSGS	6 (8.3)	11 (7.6)	0.881
IgA nephropathy	11 (15.1)	10 (6.9)	0.056
IgAVN	5 (6.8)	28 (19.4)	0.015

None of the biopsied children with MCD, post-infectious glomerulonephritis (PIGN) or tubulointerstitial nephritis (TIN) progressed to CKD 5. The median follow-up time (IQR) for patients not reaching CKD 5 was 16.2 (11.8 to 22.8) years.

By the end of 2020, 27 (4.7%) of the included patients had died, 12 of whom were in KRT (cardiac arrest and malignancy, both *n* = 3; infection, *n* = 2; stroke, gastrointestinal bleeding, accident, and refusal of further KRT, all *n* = 1). Due to lack of follow-up data in NKBR, we do not know the cause of death for patients not treated with KRT.

## Discussion

In this retrospective study on kidney biopsy findings in children and the kidney long-term outcome, MCD, IgAVN, IgA nephropathy, and FSGS accounted for nearly 50% of the biopsy diagnoses. Several other studies have also found that these four diagnoses are highly prevalent in childhood kidney biopsy [9–14]. MCD was the most common single diagnosis with no children progressing to CKD 5, whereas NPHP was as expected the most common single diagnosis associated with KRT. Our data confirm findings from other studies that MCD is a leading histology finding in kidney biopsies in children [9, 10, 15–17]. Generally, children presenting with a typical clinical picture of MCD do not need a kidney biopsy [18, 19]. The risk of later KRT in MCD is negligible with symptom-based treatment, and hence, the main challenge with this “benign” disease is the disease burden and the therapeutic challenges to avoid relapses. The high appearance of MCD reflects the incidence of the disease, the tendency of frequent relapses, steroid resistance, and the problems with nephrotoxic drugs used in the treatment. Interestingly, idiopathic FSGS and MCD may represent two histological pictures of the same podocytopathy [20, 21]. FSGS is a heterogeneous disease with many underlying causes, including more than 50 genetic mutations [21]. Some of our children with FSGS subsequently were diagnosed with various genetic mutations associated with podocyte integrity and function, predisposing for a more rapid deterioration in kidney function likely due to lower response to immunosuppressive therapy [21, 22]. Unfortunately, genetic information in the patient charts was not complete, and was thus not implemented in the present analysis. Nearly 60% of our children with FSGS reached KRT, similar to other studies [2, 23].

IgA nephropathy was the second most common primary glomerulonephritis (Fig. 1). Due to the timing, this study did not implement the Oxford classification for IgA nephropathy launched in 2009 [24]. Our analysis supports the reports from several studies that boys more often than girls develop IgA nephropathy and progress to CKD 5 [25–28].

Interestingly, some see IgA nephropathy and IgAVN as different expressions of the same disease [29–31]. Clinical course and extrarenal manifestations are necessary to differentiate IgA nephropathy and IgAVN. In 2018, Suzuki et al. suggested a shared etiology between IgA nephropathy and IgAVN when they detected glomerular galactose-deficient IgA1 in both IgA nephropathy and IgAVN, but not in other kidney diseases [32]. High serum galactose-deficient IgA1 levels are also associated with poor prognosis in IgA nephropathy [33, 34]. Based on clinical manifestations before biopsy, IgAVN represented half of the secondary systemic glomerular diseases detected in our study (Fig. 1). There was an increase in the number of kidney biopsies performed in the IgAVN group between 1990 to 1994 and 2009 to 2013. We interpret this as an increased awareness of the crucial role of biopsies in IgAVN in order to evaluate the severity of the kidney disease and implement treatment that might reduce the risk of CKD [35]. Our study shows that despite small numbers, there are similarities between IgA nephropathy and IgAVN with respect to occurrence, numbers reaching KRT and age at KRT in children with a biopsy-verified diagnosis (Table 2). The similarity in outcomes of IgA nephropathy and IgAVN in our study may be skewed by the different indications for biopsy in the two diseases: diagnosis in the former and severity and appropriateness in the latter. The actual incidence for KRT in both IgA nephropathy and IgAVN is highly uncertain as the prevalence of both diseases are uncertain and vary across the world [25, 36–40].

Clinical phenotype or genetic analyses may render a kidney biopsy redundant in many cases of kidney disease, limiting the number of such children included in kidney biopsy registries [2–5]. Collecting clinical, genetic, and pathology data in the same registry may improve our understanding of chronic kidney disease in children. Additionally, reviewing histology results in the context of genetic information may be beneficial when educating patients and their parents on disease course and management, and may indeed improve patient care [41].

The main strengths of this national study are the long follow-up time regarding progression to KRT after childhood kidney biopsy, and the fact that we could link data from two national registries both with very good attendance based on the authors’ personal experiences. It was a strength that clinicians caring for the patients retrieved clinical data at their respective hospitals. Unfortunately, the retrospective study design has several limitations including information bias, missing data, and a potential selection bias of patients selected for biopsy. Limited sample size is a general challenge in pediatric kidney disease research, and a limitation in our study was the low incidence of CKD 5.

## Conclusion

Histopathology in 50% of childhood kidney biopsies showed primary glomerulonephritis; nearly one third of these were MCD. Approximately 20% of the biopsied children had kidney diseases that progressed to CKD 5 during follow-up. NPHP was the most common diagnosis in patients later in need of KRT (100%) followed by FSGS (approximately 60%). Linking national biopsy registry data with national KRT registry data complements our understanding of the outcomes in kidney disease in childhood.

## Supplementary Information

Below is the link to the electronic supplementary material.

Graphical Abstract (PPTX 108 KB)
