# Supplementary material for: Kidney biopsy diagnosis in childhood in the Norwegian Kidney Biopsy Registry and the long-term risk of kidney replacement therapy: a 25-year follow-up
Source: Pediatr Nephrol. 2022 Aug 22;38(4):1249–56. doi: 10.1007/s00467-022-05706-y (PMC9925570; doi:10.1007/s00467-022-05706-y)
Supplement: Supplementary file 1 — Graphical Abstract (PPTX 108 KB) [file 467_2022_5706_MOESM1_ESM.pptx]

## Slide 1
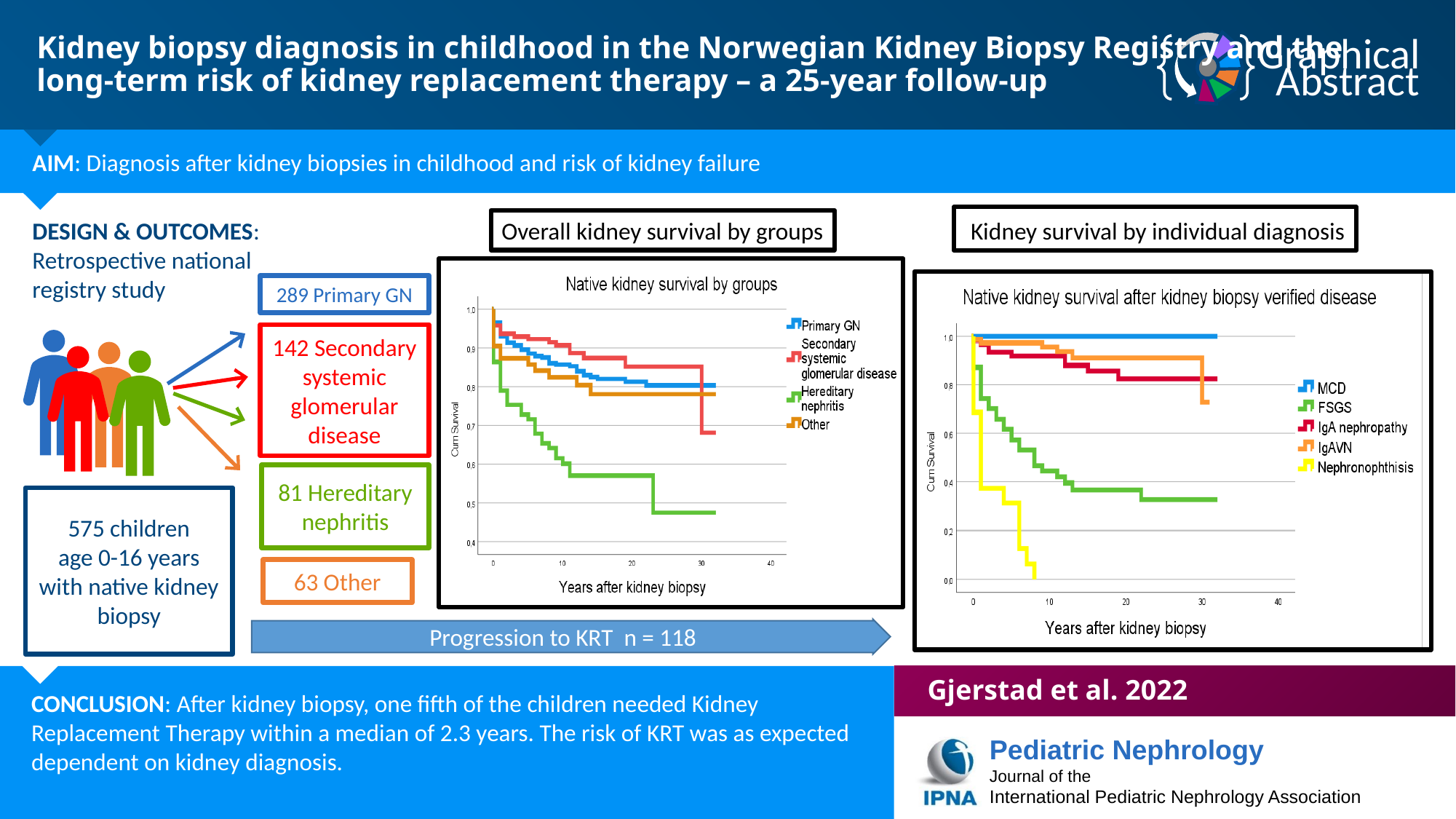

Kidney biopsy diagnosis in childhood in the Norwegian Kidney Biopsy Registry and the
long-term risk of kidney replacement therapy – a 25-year follow-up
AIM: Diagnosis after kidney biopsies in childhood and risk of kidney failure
 Kidney survival by individual diagnosis
DESIGN & OUTCOMES:
Retrospective national
registry study
Overall kidney survival by groups
289 Primary GN
142 Secondary systemic glomerular disease
81 Hereditary nephritis
575 children
age 0-16 years with native kidney biopsy
63 Other
Progression to KRT n = 118
Gjerstad et al. 2022
CONCLUSION: After kidney biopsy, one fifth of the children needed Kidney Replacement Therapy within a median of 2.3 years. The risk of KRT was as expected dependent on kidney diagnosis.
